# Editorial: Addressing the challenges in the diagnosis of depressive disorder in children and adolescents

**DOI:** 10.3389/fpsyt.2022.1011301

**Published:** 2022-11-15

**Authors:** Moon-Soo Lee, Alessandra M. Passarotti

**Affiliations:** ^1^Psychiatry/ Child and Adolescent Division, College of Medicine, Korea University, Seoul, South Korea; ^2^Department of Psychology, College of Liberal Arts and Sciences, University of Illinois Chicago, Chicago, IL, United States

**Keywords:** depressive disorder, diagnosis, comorbidities, socio-cultural aspect, follow-up, IDEA-RiSCo

The diagnosis of depression in children and adolescents is particularly challenging due to the fact that depression is quite an heterogeneous and multifactorial disorder, and, further, it often shows different clinical features in children compared to adults. Moreover, in recent years, various challenges and the impoverishment of social experiences caused by COVID-19 have further increased the prevalence of depression, especially in children and adolescents (1, 2). Therefore, reaching a better understanding of adolescent depression is currently a paramount health care goal.

Typically, clinical research studies on depression show behavioral or neural differences found in comparative studies between patient and control groups. Although these findings give us a better understanding of depression mechanisms, they are mainly based on group-level differences. Therefore, it is still difficult for clinicians to apply research findings directly to patients' diagnoses on an individual level. Despite these difficulties, it is noteworthy that important scientific advances have been made in the past couple of decades due to better clinical research and brain imaging methodologies (e.g., fMRI, DTI) that fostered research collaborations between the domains of psychiatry, clinical psychology, and cognitive and affective neuroscience. Moreover, the recent introduction of artificial intelligence represented by “machine learning”, and the concept of “precision medicine” raises the possibility of applying existing medical advances to more individualized clinical settings. This will also contribute to the objectification of the diagnosis of depressive disorders (3).

Expression of depressive disorder is influenced by developmental aspects in children and adolescents. In turn, depressive disorder greatly affects the developmental path of children and adolescents. In this Research Topic, we introduce some innovative and novel studies that address some of the diagnostic challenges in adolescent depression, as they relate to important aspects such as: clinical research supporting the diagnostic process specifically in adolescents; studying the longitudinal progression of the illness to better understand the comorbidities, risks, and protective factors involved; the development of new diagnostic tools to be used specifically with adolescent population, or the validation of existing adult measures for the adolescent population; and an in-depth examination of the neural mechanisms of adolescent depression. These challenges are addressed by the articles included in this Research Topic. As an additional strength, this Research Topic provides a unique international perspective on a depressive illness that is very sensitive to socio-cultural aspects with insights on commonalities and differences in adolescent depression world-wide.

First, with regard to improving the diagnostic process in adolescent depression, one major clinical need is that of accurately investigating associated factors with the actual status of depression. The article by Anjum et al. about depressive symptom's factors in Bangladeshi provides a new, important perspective by discussing assessment of depressive symptoms and associated risk and protective factors in adolescents in different social-cultural environments.

Second, because of the developmental aspects of depression in children and adolescents, long-term cohort observational studies are of particularly high value when trying to understand illness progression and effects of risk and protective factors. Yet, long-term follow-up studies with children and adolescents are still relatively rare. To fill this gap, the long-term follow-up study by Lai et al., “Adolescent Depression and Deliberate Self-Harm”, assessed a large sample of high school students in rural areas over 2 years, using repeated measurement data and a bivariate multilevel logistic regression model to explore the common risk and protective factors with regard to the under-studied comorbidity between depression and deliberate “self-harm”.

Third, validation of the usability of new or already validated diagnostic tools in developmental populations is another very useful goal in clinical practice. The study by Kieling et al., titled “Depression Risk-Stratified Adolescent Cohort”, deepens our understanding of risk factors in adolescent depression by assessing individual probability of depression in Brazilian adolescents using a newly developed tool for the prediction of depression risk, namely a composite risk score, the “Identifying Depression Early in Adolescence Risk Score” (IDEA-RS).

Moreover, Keller et al., in the article titled “Factor Structure of the BDI-II” provide strong evidence that the total score of the Beck Depression Inventory II (BDI-II) can be successfully used to disentangle developmental aspects from depression aspects, and to better measure depression severity in adolescents.

Finally, related to the importance of understanding the neurophysiological underpinnings and mechanisms of adolescent depression, important contributions to improving traditional diagnostic criteria have come from translational and affective neuroscience brain imaging studies aiming to identify “biobehavioral predictors of disease trajectory” (Ely et al. this issue). The review paper by Ely et al., “Reward Function in Adolescent Depression”, explains the usefulness of studying reward circuitry in adolescent depression in trans-diagnostic cohorts without being bound by categorical diagnosis. The authors review recent studies that use innovative neuroimaging techniques and integrative, data-driven analyses to examine the neural bases of “anhedonia”, used here as a clinical index of dysfunction of reward systems in the brain, along a continuum across disorders.

In conclusion, as this Research Topic of articles indicates, to meet current challenges and provide a more valid and timely diagnosis of adolescent depression, we need more interdisciplinary research between the disciplines of psychiatry, clinical psychology, developmental psychology, cognitive neuroscience and affective neuroscience. This will enable us to get more information about more detailed diagnoses, comorbidities, treatment responses and prognoses by combination of different scientific methodologies. As a future direction, we believe that the use of automated machine learning and artificial intelligence based on multimodal data and omics analysis will also help toward a more objective diagnosis of depression and quantification of key factors associated with depression.

## Author contributions

A draft was written by M-SL. The overall flow was taken and the final content was revised by AP. Both authors contributed to the article and approved the submitted version.

## Conflict of interest

The authors declare that the research was conducted in the absence of any commercial or financial relationships that could be construed as a potential conflict of interest.

## Publisher's note

All claims expressed in this article are solely those of the authors and do not necessarily represent those of their affiliated organizations, or those of the publisher, the editors and the reviewers. Any product that may be evaluated in this article, or claim that may be made by its manufacturer, is not guaranteed or endorsed by the publisher.
